# Live to Work or Love to Work: Work Craving and Work Engagement

**DOI:** 10.1371/journal.pone.0106379

**Published:** 2014-10-08

**Authors:** Kamila Wojdylo, Nicola Baumann, Lis Fischbach, Stefan Engeser

**Affiliations:** 1 Institute of Psychology, Polish Academy of Sciences, Warsaw, Poland; 2 Department of Psychology, University of Trier, Trier, Germany; Chiba University Center for Forensic Mental Health, Japan

## Abstract

**Objective:**

According to the theory of work craving, a workaholic has a craving for self-worth compensatory incentives and an expectation of relief from negative affect experienced through neurotic perfectionism and an obsessive-compulsive style of working. Research has shown that workaholism and work engagement should be considered as two distinct work styles with different health consequences. However, the mechanisms underlying the adoption of these work styles have been neglected. The present study proposes that work craving and work engagement are differentially associated with self-regulatory competencies and health. In particular, we expected that the working styles mediate the relationships between emotional self-regulation and health. Methods: In the cross-sectional study, 469 teachers from German schools completed online administered questionnaires. By means of structural equation modeling, we tested two indirect paths: a) from self-relaxation deficits via work craving to poor health and b) from self-motivation competencies via work engagement to good health.

**Results:**

As expected, we found evidence that a) the negative relationship of self-relaxation deficits on health was partially mediated by work craving and b) the positive relationship of self-motivation competencies on health was partially mediated by work engagement.

**Conclusions:**

The present study emphasizes the importance of self-regulation competencies for healthy or unhealthy work styles. Whereas work craving was associated with a low ability to down-regulate negative emotions and poor health, work engagement was associated with a high ability to up-regulate positive emotions and good health.

## Introduction

Some people (workaholics) have a strong craving for work because they only feel worthy when working hard and perfectly well whereas others (work engagers) are working hard because they enjoy work. Although some studies have shown negative relationships between workaholism and health [1], [2] or positive relationships between work engagement and health [3], [4], the self-regulatory mechanisms behind these two different work styles have been neglected so far. This research aimed at extending current knowledge in at least three respects. First, our focus on the associations between emotional self-regulation, work styles, and health outcomes enhances an understanding of the role of personal characteristics in the etiology of workaholism and its health consequences. Second, our examination of the mediation between self-regulatory competencies and health underlines the importance of work styles as mechanisms for individual well-being. Finally, our distinction between two work styles (i.e., work craving vs. work engagement) provides a differentiated picture of the relationships between work styles and health.

### Workaholism versus Work Engagement

Work engagement has been proven to be an empirically distinct construct from workaholism [5], [6]. Work engagement is defined as a positive, fullfilling, work-related state of mind characterized by vigor (energy, concentration, strain, persistence in the face of inconviniencies), dedication (inspiration and challenge, full of work enthusiasm) and absorption (like an enduring flow-experience) [7]. Although the concept of work engagement was clearly defined, the concept of workaholism remained vague because of two reasons. First, work engagement is still considered by some scholars as a possible dimension of workaholism (e.g., [8]). Second, recent conceptualisations of workaholism propose obsessive inner drive as the core characteristic of workaholism and neglect its addictive nature (e.g., [7]). To avoid the vagueness of the concept of “workaholism”, we explicitly differentiate between work engagement and work addiction. Thus, in the present study, we adopted the conceptualization of workaholism as work craving [9], [10].

Wojdylo [9] proposed the theory of work addiction, which she called work craving. She argued that the core characteristics of work addiction are not fully explained by obsessive inner drive but constitute other addictive mechanisms. In her view, a main mechanism of work addiction is the compensatory function of emotions, which explains the inner obsessive drive of workaholics in fulfilling unrealistic standards of perfectionism. Thus, in contrast to previous theories of workaholism (e.g. [11], [7], [8]), the conceptualization of work craving constitutes a synthesis of obsessive-compulsive and *addictive* elements. Specifically, in addition to the compulsive/behavioral component, work craving theory includes two hedonic/affective components (anticipation of self-worth compensation and anticipation of reduction of negative affect or withdrawal symptoms resulting from working), and a learning component (neurotic perfectionism) [9], [10]. Thus, work craving is defined as an emotional-motivational state oriented at compensation of negative emotions through an obsessive-compulsive work style and a desire for unrealistic (neurotic) perfectionistic standards.

Studies have shown that work engagement and workaholism have distinct regulatory mechanisms and outcomes. Workaholism and work engagement share the behavioral component (working excessively hard, high work involvement), but the emotional and motivational aspects of these phenomena differ fundamentally. Whereas workaholics are motivated by an obsessive inner drive they cannot resist, engaged employees are intrinsically motivated, have a sense of energetic and effective connection with their work activities, and view themselves as able to deal well with the demands of their jobs [12]. These findings imply that workaholism and work engagement are differently related with self-regulation competencies (i.e., self-relaxation and self-motivation). According to work craving theory, the emotion-regulatory components inherent in work craving (i.e., anticipation of reduction of negative emotions and desire for self-worth compensatory incentives) imply that work craving should derive from low rather than high self-regulatory competencies [10].

As mentioned above, studies have also shown that workaholism and work engagement are related with distinct outcomes. Work engagement is related to desirable outcomes like job and life satisfaction, better job performance and negatively associated with ill being [13], [14]). Workaholism, in contrast, is related to negative outcomes like psychosomatic symptoms, mental and physical health complaints [15], [2], [16], [14], [10], poor emotional well-being [17], increased work-family conflict [18], and low life satisfaction [19], [20]). In the present study, we therefore expected work craving to be negatively associated with health (*H1a*) and work engagement to be positively associated with health (*H1b*).

### Emotional Self-Regulation and Well-Being

The ability to self-regulate one's feelings and thoughts plays an important role in personality functioning [21], [22] and can be differentiated into two major self-regulation competencies: self-relaxation and self-motivation [23], [24], [25]. Individual differences in self-relaxation and self-motivation competencies can be assessed by the failure- and decision-related action control scales, respectively [26]. In these scales, high levels of self-regulation competencies are called *action orientation* whereas low levels are called *state orientation* and indicate a low action orientation.

Failure-related action orientation (AOF) is the ability to reduce the negative affect during or after confrontation with failure or threat (high self-relaxation) and to keep up or even enhance access to the self (i.e., implicit representations of own wishes, goals, and preferences). Thus, it promotes the formation of realistic and self-congruent goals and, in turn, well-being especially under threatening conditions (e.g., [27], [28], [29]). In contrast, failure-related state orientation (the low end of the AOF scale) denotes a low ability to self-regulate negative affect (low self-relaxation) and is characterized by ruminative thoughts. Studies found relationships between failure-related state orientation and psychosomatic symptoms, depression, low self-esteem, and self-consciousness [27], [30], [31] as well as workaholism [32].

Decision-related action orientation (AOD) is the ability to up-regulate positive affect, to overcome feelings of listlessness, and to foster confidence and enthusiasm despite the presence of challenging demands and difficulties (high self-motivation). Thus, it is a decisive precondition for an efficient translation of intentions into action. In contrast, decision-related state orientation (the low end of the AOD scale) denotes a low ability to self-generate positive affect (low self-motivation) and it characterized by hesitation. Studies have linked decision-related state orientation to reduced well-being [27], a low ability to behave according to one's preferences [33], procrastination [34] and workaholism [32].

Hence, we hypothesize that self-relaxation and self- motivation competencies are negatively associated with work craving (*H2a*) and positively with work engagement (*H2b*). Furthermore, we hypothesize that self-relaxation and self-motivation competencies are positively related with health indices (*H3*). Finally, we consider work styles as mediators between self-regulation competencies and health. Despite the correlational nature of many of the reviewed findings, self-regulation competencies are better conceived of as antecedents rather than consequences of work styles and health because failure- and decision-related action orientation are personality dispositions that develop during early childhood and are rather stable over time [25], [26]. In addition, longitudinal findings indicate that work styles precede rather than follow from health states [14]. We hypothesize that the relationship between self-relaxation and self-motivation competencies and good health is partially mediated by work engagement (*H4a*), and that the relationship between self-relaxation deficits and self-motivation deficits and poor health is partially mediated by work craving (*H4b*).

### Work Craving, Work Engagement, and Working Hours

According to the view of lay people, working a lot can be equated to workaholism. From our point of view, working hours and workaholism have to be separated. Wojdylo et al. [10] empirically showed that work cravers and work engagers did indeed work an equal number of hours. Thus, differences between work craving and work engagement cannot be attributed to different hours of working but, instead, to different work styles alone. We aspired to replicate these findings, so we also explored the relationship between working hours and the two different work styles of work craving and work engagement. We hypothesize that the relationship between work craving and number of worked hours is low (*H5*).

## Method

We decided to conduct our study in a sample of German school-teachers for two reasons. First, differences between state- and action-oriented individuals are typically observed under demanding conditions. We expected the workplace of teachers to be sufficiently demanding to necessitate the use of self-regulation competencies (because of high stress conditions and emotional demands) and, therefore, to observe a benefit of action orientation. Second, because of the high work-related stress, burnout rates, and mental health problems among teachers in Germany [35], we also expected to find sufficient levels of health complaints and unhealthy work style. When testing at schools in Germany, we needed to go through an ethical board of the respective federal state coordinating assessments at schools (ADD: Aufsichts- und Dienstleistungsdirektion). The ADD of the federal state of Rheinland-Pfalz approved our study (ADD approval 51 111-31/129-12). In addition, the representative for data security approved our study (see attachment: Approval 6.08.22.001:0363). Participants were given detailed information about their rights and gave their informed consent through participation in the study. Participants have provided their written informed consent to participate in this study. Only data from participants were used who fully completed the study and did not send an e-mail to the study coordinator to refrain from the study.

### Participants

The present study is a part of the Work Craving International Project (WCIP), a large cohort research realized in Poland and Germany (“Work craving – personality antecedents and regulatory mechanisms”). The WCIP aims at examining the new conceptualization of workaholism as work craving and its personality mechanisms [10].

Four hundred and sixty-nine teachers from the Federal State Rheinland-Pfalz of Germany participated in the present study (345 women and 121 men, 3 participants gave no information on gender). Participants were recruited via e-mail to all school principals across Rheinland-Pfalz. The principles were asked to distribute the information among the staff. Participants completed the questionnaire voluntarily online which took about 45 minutes. Participants' average age was 45 years (SD  =  10.57), with a range of 21 to 64 years. 62.5 % were married, 20.5 % were living in a relationship, 14.9 % were living alone, and 1.9 % did not provide information about their marital status. 36.7 % were teaching in primary schools, 14.7 % in secondary/graduate schools, 16.2 % in special schools, 15.6 % in vocational schools, and 16.8% in other school forms. 63.1 % were employed full-time, 17.3 % worked 30 hours a week, 11.7 % were employed part-time, 6 % worked less than part-time, and 1.9 % did not give information regarding their employment.

### Measures and Procedure

All questionnaires were administered online in a German version.

#### Work engagement

The Utrecht Work Engagement Scale (UWES, [36]) was used. The UWES consists of 17 items and has three subscales: Vigor (*“At my work, I feel bursting with energy”*), dedication, (*“I find the work that I do full of meaning and purpose”*), and absorption (*“When I am working, I forget everything else around me”*). Items were scored by means of a 7-point frequency rating scale ranging from 0 (never) to 6 (each day). We considered higher general scores as an indicator of higher work engagement. The internal consistency (Cronbach's Alpha) of the UWES was α  = .90.

#### Work craving

The German version of 28-item Work Craving Scale (WCS, [10]) was used to assess workaholism. The WCS has four subscales (7 items each): Obsessive-compulsive desire for work (*“I have an urge to work more and more”*), anticipation of self-worth compensatory incentives from work (*“Overworking makes me feel important”*), anticipation of reduction of negative affect and withdrawal symptoms (*“Working now would bring me a relief”*), *and* neurotic perfectionism (*“Even though I perform a task very carefully, I feel that it is done not correctly enough”*). Items were scored by means of a 7-point agreement rating scale ranging from 1 (not at all) to 7 (completely). In our study, higher general scores were taken to indicate higher work craving (α  = .95).

#### Emotional self-regulation

Self-regulation competencies were assessed by the Action Control Scale (ACS, [37]). The failure-related dimension of action orientation (AOF) was used to assess the ability to down-regulate negative affect following a failure, thus self-relaxation. An example item for AOF is *“I've worked for weeks on one project and then everything goes completely wrong: (a) It takes me a long time to get over it, or (b) It bothers me for a while, but then I don't think about it anymore”*. The decision-related dimension of action orientation (AOD) was used to assess the ability to up-regulate positive affect preceding an action, thus self-motivation. An example item for AOD is “*When I am getting ready to tackle a difficult problem: (a) It feels like I am facing a big mountain that I don't think I can climb,or (b) I look for a way that the problem can be approached in a suitable manner.”* In the example items, options "a" reflect the state-oriented response alternatives and options "b" the action-oriented response alternatives. Each dimension of the scale consists of 12 items. For each dimension, the action-oriented response alternatives were summed so that each scale ranged from 0 to 12, with lower scores indicating lower action orientation (i.e. state orientation) and higher scores indicating higher action orientation. For the AOF dimension α  = .84 and for the AOD dimension α  = .87.

#### General health

The General Health Questionnaire (GHQ-28, [38]) was used to assess the mental health status. The GHQ has four subscales: Somatic symptoms (*“Have you recently felt that you are ill?”*), anxiety and insomnia (*“Have you recently lost much sleep over worry?”*), social dysfunction (*“Have you recently been taking longer over the things you do?”*), and severe depression (*“Have you recently felt that life is not worth living?”*) with 7-items each. All 28 items were rated on four-point scales from 1 to 4 with slightly different labels across items. Items' polarity was reversed so that higher GHQ values correspond to better general mental health. In the following analyses, only a general score was considered (α  = .94).

#### Working hours

Spent working hours was assessed by one item. Participants were asked to estimate the average daily hours they spend working: *“How many hours do you actually work on an average day?”*

## Results

### Descriptives and correlations

Table 1 presents an overview of the descriptive results and correlations for the study variables. As expected, work craving correlated negatively (*H1a*) and work engagement positively with health (*H1b*). Second, self-regulatory competencies (AOF and AOD) correlated negatively with work craving (*H2a*) and positively with work engagement (*H2b*). Third, self-relaxation (AOF) and self-motivation (AOD) showed significantly positive correlations with health (*H3*) and with each other. Contrary to previous findings [10], there was a significant relationship between work craving and working hours, but the relationship was very weak (*r*  = .10) indicating that work craving cannot be inferred from working hours (*H5*). Finally, in compliance with previous findings [10], the correlation between work engagement and work craving was very small indicating that they are distinct work styles.

**Table 1:** Descriptives and Bivariate Correlations (Pearson) Between Study Variables (N  =  469).

	*M*	*SD*	Scale	Range	(2)	(3)	(4)	(5)	(6)	(7)	Gender ^a^
(1) Action Orientation (AOF)	5.96	3.46	0 – 12	0 – 12	.52***	.24***	−.53***	.50***	.08	.05	.22***
(2) Action Orientation (AOD)	7.54	3.66	0 – 12	0 – 12		.34***	−.31***	.41***	.03	.03	.09
(3) Work Engagement (UWES)	5.64	0.84	1 – 7	2.2 – 7.0			.02	.32***	.08	.02	−.12*
(4) Work Craving (WCS)	2.78	1.09	1 – 7	1.0 – 6.4				−.48***	.10*	−.12*	−.08*
(5) General Health (GHQ)	3.12	0.45	1 – 4	1.0 – 3.9					−.06	.08	.07
(6) Working Hours ^b^	7.68	2.36	0 – 24	1.0 – 15						.00	.15**
(7) Age ^c^	44.94	10.57		21 – 64							.19***

*Note.* ^a^female  =  1; male  =  2. ^b^ *N*  =  436. ^c^ *N*  =  459.

* *p* <.05 ** *p* <.01 *** *p* <.001.

### Structural equation modeling

**Figure 1:**
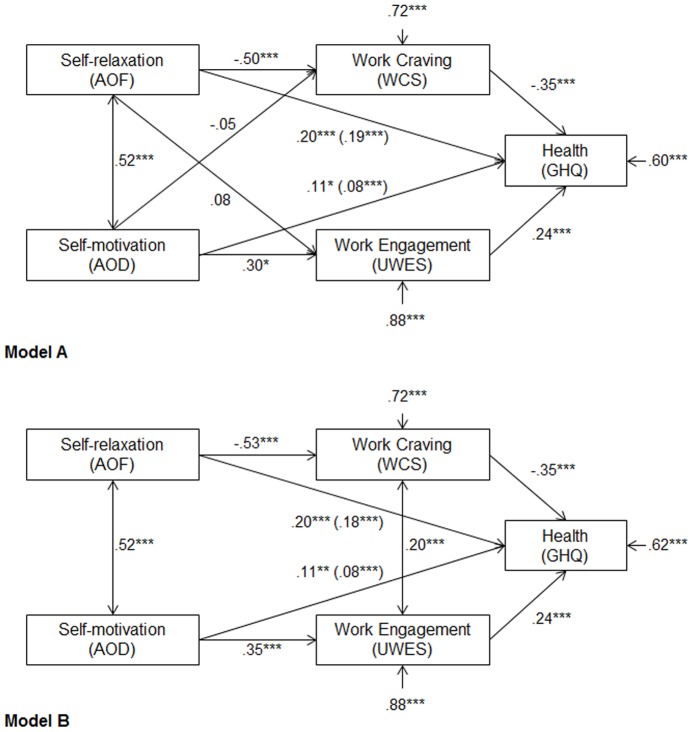
Regression coefficients of two path models tested through structural equation modeling. Indirect path coefficients are in parentheses. The residual variance components (error variances) indicate the amount of unexplained variance. For each observed variable, R^2^  =  (1 - error variance). * p <.05 *** p <.001.

To further test our hypotheses regarding meditational effects (*H4a* and *H4b*), we used structural equation modeling (SEM). We designed a path-model (Figure 1, Model A) allowing us to test the four mediation assumptions at the same time: that work craving mediates between self-relaxation deficits and poor health and between self-motivation deficits and poor health whereas work engagement mediates between self-relaxation competencies and good health and between self-motivation competencies and good health. The Model Fit was estimated by means of Mplus6 [39]. The regression coefficients were adjoined to the hypothetical Model A in Figure 1. For Model A, good fit indices were achieved (CFI  = .96; SRMR  = .04; χ^2^(1)  =  17.997). The cut off ranges for the absolute fit indices were achieved (cf. [40], [41]). Looking at the path coefficients of Model A, work craving was associated with lower general health whereas AOD, AOF, and work engagement were associated with higher general health. Most important for our theoretical expectations, only the paths from AOF to work craving and from AOD to work engagement were significant, whereas the paths from AOF to work engagement and from AOD to work craving were low and not significant.

According to these results, we tested an alternative model (Figure 1, Model B) with only two indirect paths at the same time: that work craving mediates between self-relaxation deficits and poor health whereas work engagement mediates between self-motivation competencies and good health. Model B reached with a CFI  = .997, SRMR  = .019, and χ^2^(2)  =  3.27 a generally good fit. We found significant direct effects from AOF and AOD on health (*β_AOF_*  = .20, *p* <.01; *β_AOD_*  = .11, *p* <.01). Consistent with expectations, we also found significant indirect effects from AOF through work craving on health (*H4a*: *β_AOF_WCS_*  =  -.19, *p* <.05) and from AOD through work engagement on health (*H4b*: *β_AOD_UWES_*  =  −.08, *p* <.05).

When comparing the model fit of the competing models A and B, Model B reached a better fit. According to the chi square difference test, the difference between the models was significant (Δχ^2^(1)  =  14.72, *p* <.001. The findings indicated that the relationship between self-relaxation deficits (but not by self-motivation deficits) and poor health was partially mediated by work craving whereas the relationship between self-motivation competencies (but not self-relaxation competencies) and good health was partially mediated by work engagement.

## Discussion

The aim of the present paper was to understand more precisely the differences between work craving and work engagement, their regulatory mechanisms, and outcomes. Our study showed that work cravers have low abilities in self-relaxation as well as low abilities in self-motivation, whereas work engagers have high self-regulatory abilities of both types. These findings are consistent with and extent findings regarding motivational differences between workaholics and engaged employees. Van Beek, Hu, Schaufeli, Taris, and Schreurs [42], for example, found that workaholic employees feel motivated by instrumental values and tend to introject goals whereas engaged employees work not just for the instrumental value, but also value the work itself and integrate goals into the self (see also: [43]). These motivational differences can be explained by the presently observed differences in self-regulation competencies between work cravers and work engagers. According to Kuhl [24], [25] self-regulation competencies form the basis for the creation of realistic, self-congruent goals and self-determination [27], [28], [29]. Thus, motivational differences between workaholics (motivated by introjected goals) and work engagers (motivated by integrated goals) can be explained by self-regulatory deficits and self-regulatory competencies. This notion, of course, requires father exploration in the next study.

The present study supported our assumption that work craving and work engagement are partial mediators between self-regulatory competencies and health symptoms. Interestingly, although both types of self-regulatory abilities were associated with more healthy and less unhealthy work styles, results indicated distinct mediating roles of work engagement and work craving. High work craving partially mediated the relationship between self-relaxation deficits (but not self-motivation deficits) and poor health whereas high work engagement partially mediated the relationship between self-motivation competencies (but not self-relaxation competencies) and good health.

Work craving, on the one hand, was fueled in our study in particular by a low ability to self-regulate negative emotions (low self-relaxation). This finding is consistent with Kuhl's [25], [44] theorizing that overworking may be a coping strategy that suppresses negative feelings without truly self-regulating them. Work cravers' self-relaxation deficits indicate reduced access to the self that might express itself in their tendency to set unrealistically high standards of achievement. Because workaholics do not completely lack self-motivation and/or use external sources of motivation (cf. [42]), they are able to become initiative and strive for their overly high standards of excellence. Unreachable standards keep them busy and distracted from negative feelings. However, distraction through overworking does not solve any problems or promote success because workaholics' goals are unrealistically high [45] and introjected rather than integrated into the self [42], [43]. Thus, work craving may instigate a vicious “loss-of-autonomy” cycle [33] in which the self is suppressed, conflict accumulated, effortful control increased, and the self even further suppressed.

Work engagement, on the other hand, was associated in our study in particular with a high ability to self-regulate positive emotions (high self-motivation). Work engagers' high self-motivation competencies make it easier to bring up the positive affect needed to translate an intention into action and contribute to an identification with their goals [42]. They know how they can please themselves and savor the immediate hedonic affect inherent in working. In an experience sampling study by Bledow, Schmitt, Frese, and Kühnel [46], work engagement was strongest among employees who had a negative onset and managed to shift to a positive mood throughout their working day. Consistent with our findings, these affective shifts were supported by personal resources (i.e., dispositional affectivity) related to high positive rather than low negative affect. Our findings extent the work by Bledow et al. [46] by showing that personal resources for work engagement are not restricted to *affect sensitivity* (i.e., how easily one *enters* an emotional state) but also available at the level of *affect regulation* (i.e., how easily one *leaves* and actively changes an emotional state once it is aroused) [23]. This is informative because people's affect sensitivity is more genetically predisposed and less malleable through training than their affect regulation competencies (cf. [25]).

To summarize, the present results revealed that work craving and work engagement have distinct self-regulatory underpinnings and opposing effects on health. Despite the high correlation, self-relaxation and self-motivation competencies show functional dissociations: whereas self-relaxation competencies obviate work craving, self-motivation competencies abet work engagement. These specific effects on two distinct work styles are part of the reasons why self-relaxation and self-motivation contribute to employees' general health.

Furthermore, in earlier research on workaholism, hard workers who worked long hours while experiencing an inner drive were branded workaholics. For a long time, workaholism was defined and measured as excessive working, that is, by quantity (e.g., [47]). Our results replicated the finding by Wojdylo et al. [10] that work craving is only weakly correlated with and cannot be reduced to spending more time at work.

### Limitations and Future Perspectives

The main limitation of the current study is its cross-sectional nature that precludes cause–effect relationships being uncovered. Emotional self-regulation competencies are usually considered as relative stable personality dispositions that develop in early childhood [24], [25]. Therefore, it is unlikely that the scores on our self-regulation measures are caused by work craving/work engagement or health. In contrast, the causal direction of the association between health and work craving/work engagement is less clear. Although previous research longitudinally established that high workaholism preceded poor health and high work engagement preceded good health [14], we cannot exclude the possibility that unhealthy workers display more workaholic behaviors and healthy workers display more enthusiastic behaviors. These notions can only be tested adequately using a longitudinal design.

Second, the current data set was drawn from a very specific group of workers (i.e., teachers). Although this particular group has frequently been assumed to be a high-risk group for health problems [48], [49], it should be noted that the fact that all participants belonged to this high-risk group could well have led to a restriction in the variance of the study variables. The unique nature of the present sample underlines the need to replicate the current findings on samples of workers from different occupations.

Third, in the present studies, only self-reports were used to measure the constructs. Thus, we cannot preclude an impact of social desirability on the data. For instance, respondents could try to minimize or maximize their problems by under- or over-reporting the severity and the frequency of symptoms. Workaholics might also deny their condition in the same way as people suffering from alcoholism deny drinking. Thus, our present findings should be validated with more objective measures or with additional ratings from family members regarding criteria for work craving.

### Practical Implications

As for the practical implications of the present study, it is informative to see that not only current motivational states such as controlled/autonomous motivation [43] or introjected/identified motivation (cf. [42]) but also individual differences in the ability to self-regulate emotions (i.e., action vs. state orientation) are strongly associated with workaholism and work engagement. The role of self-regulation in the etiology of work craving/work engagement has received less attention than would be warranted when considering the strength of the relationships. Especially a lack of self-relaxation was strongly associated with work craving, suggesting that the concepts of self-regulatory competencies may be a good starting point for interventions at the individual and organizational level to prevent and to treat workaholism. The theory and findings on self-regulation can help to further understand the causes of workaholic behavior, while psychotherapeutic techniques - especially those focused on working with emotions, like emotion-focused therapy [50], introvision methods [51], or schema-focused therapy [52] - may be used in assisting workaholic clients to overcome their self-regulatory deficits and internal conflicts.

In conclusion, the present findings may contribute to the development of effective interventions that may help people suffering from work craving to reduce their tendency to “live to work” and promote their ability to “love to work”. The fact that work craving and work engagement exhibit different patterns of possible causes and consequences implies that different intervention strategies should be used when work craving is to be reduced or work engagement is to be enhanced.
